# An Overview of Antibiotic Therapy for Early- and Late-Onset Neonatal Sepsis: Current Strategies and Future Prospects

**DOI:** 10.3390/antibiotics13030250

**Published:** 2024-03-10

**Authors:** Giovanni Boscarino, Rossana Romano, Carlotta Iotti, Francesca Tegoni, Serafina Perrone, Susanna Esposito

**Affiliations:** 1 Pediatric Clinic, University Hospital, Department of Medicine and Surgery, University of Parma, 43126 Parma, Italy; giovanni.boscarino@unipr.it (G.B.); rossana.romano@unipr.it (R.R.); carlotta.iotti@unipr.it (C.I.); francesca.tegoni@unipr.it (F.T.); 2 PNeonatology Unit, University Hospital, Department of Medicine and Surgery, University of Parma, 43126 Parma, Italy; serafina.perrone@unipr.it

**Keywords:** antibiotic therapy, antimicrobial resistance, neonatal sepsis, early-onset sepsis, late-onset sepsis

## Abstract

Neonatal sepsis is a clinical syndrome mainly associated with a bacterial infection leading to severe clinical manifestations that could be associated with fatal sequalae. According to the time of onset, neonatal sepsis is categorized as early- (EOS) or late-onset sepsis (LOS). Despite blood culture being the gold standard for diagnosis, it has several limitations, and early diagnosis is not immediate. Consequently, most infants who start empirical antimicrobial therapy do not have an underlying infection. Despite stewardship programs partially reduced this negative trend, in neonatology, antibiotic overuse still persists, and it is associated with several relevant problems, the first of which is the increase in antimicrobial resistance (AMR). Starting with these considerations, we performed a narrative review to summarize the main findings and the future prospects regarding antibiotics use to treat neonatal sepsis. Because of the impact on morbidity and mortality that EOS and LOS entail, it is essential to start an effective and prompt treatment as soon as possible. The use of targeted antibiotics is peremptory as soon as the pathogen in the culture is detected. Although prompt therapy is essential, it should be better assessed whether, when and how to treat neonates with antibiotics, even those at higher risk. Considering that we are certainly in the worrying era defined as the “post-antibiotic era”, it is still essential and urgent to define novel strategies for the development of antibacterial compounds with new targets or mechanisms of action. A future strategy could also be to perform well-designed studies to develop innovative algorithms for improving the etiological diagnosis of infection, allowing for more personalized use of the antibiotics to treat EOS and LOS.

## 1. Introduction

Neonatal sepsis is a clinical syndrome associated with a bacterial infection leading to severe clinical manifestations, such as hemodynamic instability or other systemic manifestation, that could be associated with fatal sequalae [1,2]. Death is an unfavorable outcome in the presence of severe disease (24%) in low–middle-income countries (LMICs), while in industrialized countries, it occurs in 3–4% of newborns [1,2]. However, among the survivors, a higher risk of adverse neurological and growth long-term outcomes has been reported [3]. Fleischmann et al. performed a systematic review and meta-analysis in order to increase the data inputs from LMICs, showing that neonatal sepsis is common and often fatal in LMICs, although it incidence remains unknown in most of these countries [4].

**Figure 1:**
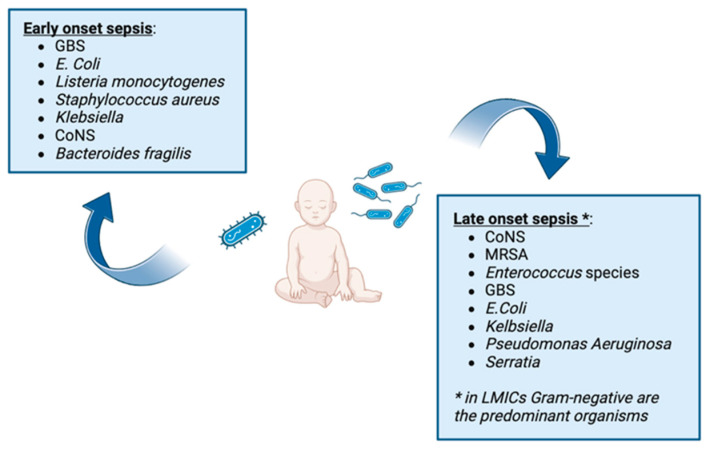
Major bacterial species associated with neonatal sepsis.

According to the time of onset, neonatal sepsis is categorized as early- or late-onset sepsis [4]. Early-onset sepsis (EOS) is considered when clinical manifestations start in the first 72 h of life. However, some authors define EOS as sepsis occurring even within 8 days after birth [5], highlighting the need to harmonize this definition. It is mainly considered as a partum or peripartum vertical transmitted infection, starting in the maternal genitourinary tract. The major bacterial species associated with neonatal sepsis are represented in Figure 1.

GBS (Group B *Streptococcus*); CoNS (coagulase-negative *staphylococci*); MRSA (methicillin-resistant *Staphylococcus aureus*); LMICs (low–middle-income countries). 

The most frequent bacteria involved in EOS are Group B *Streptococcus* (GBS—43%) and *Escherichia coli* (29%) [6]. The main risk factors for EOS are maternal chorioamnionitis, an amniotic membrane rupture lasting for more than 18 h and untreated GBS colonization. Despite the majority of the guidelines defining EOS as an infection that manifested within the first 72 h of life, an infection caused by GBS, despite the perinatal etiology, could occur within the first 7 days of life [6]. Late-onset sepsis (LOS) is usually defined as an infection that occurs after the first 72 h of life; it is associated with infants who remain hospitalized for a long time and/or with a compromised immune system. As previously reported, some authors considered LOS as sepsis occurring 8 days after being born [5]. It is associated with preterm babies, or full-term newborns that required prolonged ventilation or invasive procedures, and the microorganism of LOS are pathogens acquired in a hospital setting. The other important risk factors for LOS are the administration of H_2_ blockers, prolonged empirical early antibiotic therapy, catheters, and skin lesions [7,8,9]. It is important to underline that LOS can manifest also after discharge. The most common pathogens in high-income countries are Gram-positive organisms, including coagulase-negative staphylococci (CoNS) and streptococci [4]. In LMICs, Gram-negative bacteria are the predominant organisms [10,11,12].

The early diagnosis of neonatal sepsis is not immediate. Blood culture is the gold standard for diagnosis, but has several limitations, mainly the low sensitivity and the long turnaround time that contribute to inappropriate antibiotic therapy [13]. Nonculture-based techniques, including molecular methods and mass spectrometry, may overcome some of the limitations seen with culture-based techniques [14,15]. Biomarkers, including hematological indices, cell adhesion molecules, interleukins, and acute-phase reactants, have been used for the diagnosis of neonatal sepsis [14,15]. However, the search for an ideal biomarker that has adequate diagnostic accuracy early on is still ongoing [14,15]. Thus, all neonates at risk of infection, including preterm infants, are treated with large-spectrum empirical antibiotics, even in the absence of a clinical manifestation [16]. Despite stewardship programs partially reducing this negative trend [17,18], in neonatal intensive care units (NICUs), antibiotic overuse as a strategy of prevention still persists, and it is associated with several relevant problems, the first of which is an increase in antimicrobial resistance (AMR) [19,20,21].

Over the last year, some adjunctive therapies to treat or prevent neonatal sepsis have been proposed, but none of the these to date can be considered a definitive advance [22,23], and antibiotics remain the gold standard drug to treat sepsis. Clinically, there is often little difference between sepsis that is caused by an identified pathogen and sepsis that is caused by an unknown pathogen [6]. With the widespread use of antibiotics, AMR has become more serious problem, and we have entered in the “post-antibiotic era”, with the need for new antibiotics to treat bacterial infections [24,25,26]. Starting with these considerations, we aimed to perform a narrative review to summarize the main findings and the future prospects about the antibiotics used to treat neonatal sepsis, both EOS and LOS. The MEDLINE–PubMed database was searched to collect and select publications from 2000 to 2023. The search included randomized placebo-controlled trials, controlled clinical trials, double-blind, randomized controlled studies and systematic reviews. We performed electronic research on the PubMed/MEDLINE database using “neonatal sepsis” or “EOS” OR “LOS” OR “neonatal sepsis” OR “early onset sepsis” OR “late onset sepsis” AND “antibiotic” OR “treatment” as Mesh terms. We selected only English published manuscript.

## 2. Early-Onset Sepsis

In EOS, early diagnosis is essential because the outcome depends on the timeliness of antibiotic therapy, especially for preterm newborns or those who are small for their gestational age (GA) considering the immaturity of their immune system [27,28]. The risk of EOS is inversely related to GA, with the highest rates occurring between 22 weeks and 28 weeks of GA [29]. The aims of the recent guidelines are to identify infected newborns early, to treat them adequately and to minimize the use of antibiotics [30,31]. The diagnosis of EOS can be exceedingly difficult based solely on the clinical findings. Despite blood culture still being considered the gold standard for diagnosis, it has several limitations and needs from 2 to 7 days for the laboratory results to be retrieved. Unfortunately, none of the studied biomarkers that have been proposed fulfill all the criteria for becoming an ideal marker [15].

The National Institute for Health and Care Excellence (NICE) guidelines use “red flags” and “non-red flags”, considering risk factors and clinical findings, to identify newborns that require antibiotic treatment (Table 1) [32].

In babies with one “red flag” or more than two “non-red flags”, it is recommended to start antibiotic treatment after a blood culture has been performed. In case of the absence of “red flags” and only one “non-red flag”, clinical judgement should be used.

The American Pediatric guidelines also suggest a strategy to treat or not treat the babies based on a “sepsis calculator” that considers specific pre- or post-natal variables that help to estimate the risk of sepsis [27]. In most cases, if the neonate remains asymptomatic and the cultures are still negative between 48 and 72 h, the suspension of antibiotic treatment is recommended [33,34].

It has been demonstrated that the prolonged overuse of antibiotics increases the risk of mortality, necrotizing enterocolitis, bronchopulmonary dysplasia, fungal infections and, overall, AMR [10,35,36,37,38,39]. These findings could be due to the administration of antibiotics by physicians to the sickest infants. The other potential mechanisms include dysbiosis that alters the interactions between colonizing flora in supporting health and promoting immunity [40]. Dysbiosis due to the early and prolonged use of antibiotics has been associated with long-term health problems, such as obesity, metabolic diseases, asthma, inflammatory bowel diseases, atopic manifestations and autism spectrum disorder [37,41,42,43,44].

For EOS, the actual guidelines suggest Ampicillin or Gentamicin as the first-choice empirical therapy [27]. Third-generation Cephalosporines represent an alternative to aminoglycosides, even though they are mainly eliminated by the kidneys, and their elimination rates are reduced at birth due to the infant’s reduced renal maturation [45]. Other studies have reported the rapid development of resistance for this class of antibiotics, and the prolonged use of third-generation Cephalosporins increases the risk of invasive candidiasis [35,46]. However, when meningitis is suspected, they represent the first choice for penetration into the cerebrospinal fluid [32]. Cefotaxime is recommended as a first-line Cephalosporin because Ceftriaxone is contraindicated in neonates due to it is highly protein-bound nature that may displace bilirubin from the albumin-binding sites, causing a higher bilirubin-free concentration with subsequent accumulation in the tissues, increasing the risk of kernicterus [47]. It is important to also underline the dangerous interaction between Ceftriaxone and calcium that could induce the precipitation of calcium, causing serious adverse events such as embolism [48]. Additional antibiotic therapy should be guided by local AMR and epidemiological data. The duration of therapy should depend on the results of cultural analyses, which are performed before starting therapy, and the neonates’ health conditions.

The pathogens responsible for EOS differ widely depending on the socio-economic conditions of different countries [49]. The pathogens most frequently isolated in high-income countries are GBS (decreasing thanks to progress in the control of maternal infections) and *Escherichia coli* [50,51]. Instead, in LMICs, these were the first-place Gram-negative bacteria Enterobacteriaceae (i.e., *Klebsiella*) and Gram-positive bacteria such as *Staphylococcus aureus* and CoNS [52]. However, in both areas, the increase in AMR represents an emerging crucial problem [53]. Indeed, also, in high-income countries, despite the combination of Ampicillin + Gentamicin being appropriate, cases of resistant *E. coli* are increasing [54]. In addition, it has been reported in surveillance studies that up to 2% of *E. coli* cases are resistant to both Ampicillin and Gentamicin, and that *Bacteroides fragilis* is not uniformly sensitive to these drugs [55]. Thus, the empirical addition of broader-spectrum antibiotics could be considered only in extremely preterm infants at higher risk until the results of a culture have been obtained. Berardi et al. performed a retrospective study to supply Italian data on the antimicrobial susceptibility of EOS pathogens [56]. They found that 2/3 of the *E. coli* isolates were resistant to Ampicillin and to Gentamicin [56]. Considering the increased rate of EOS caused by *E. coli,* these data are relevant for the choice of empirical therapy. In addition, the authors found that the *E. coli* tested were susceptible to Amikacin, suggesting that Gentamicin could replace this for empirical therapy in selected cases, such as high-risk preterm neonates [56].

Encouraging data were reported by Flannery et al., who proved that most bacteria are susceptible to the combination of Ampicillin + Gentamicin [54]. The authors also stated that the non-susceptibility rates of *E. coli* to Ampicillin and Gentamicin were 77.8% and 10%, respectively, and 8.9% for both. However, in this study, the authors did not find resistance to carbapenems [54].

In LMICs, other studies highlighted the resistance or reduced sensitivity to the World Health Organization (WHO)-recommended first- and second-line empirical antibiotics for Gram-negative bacteria, which are a worrying cause of neonatal sepsis in LMICs [57]. In addition, alarming data deriving from South Africa showed a significant increase in multi-drug-resistant (MDR) *Enterobacteriaceae* in neonatal sepsis [58]. This confirms the urgent need to intensify antimicrobial stewardship and prevention in neonatal units, especially in LMICs. Indeed, if broad-spectrum antibiotics guarantee greater coverage and safety in the first phase of therapy when the culture results are not available, their prolonged use is associated with a greater development of resistance [58]. Thus, the indication to shift towards more narrow-spectrum antibiotics as soon as the microbiological results become available still is peremptory [59,60].

A drug that could play a key role in MDR Gram-negative infections is Colistin, a molecule with extremely effective action against this class of bacteria, which was previously excluded due to collateral nephrotoxicity [59]. Ambreen et al. demonstrated positive effects of Colistin in a neonatal population of MDR sepsis in Pakistan with a moderate frequency of related adverse effects (nephrotoxicity: 5.2%; seizures: 13.7%; electrolyte imbalance: 18.3%) [61].

In vitro studies are testing new combinations of antibiotics, such as Fosfomycin + Amikacin or Flomoxef + Amikacin, which would guarantee a synergistic effect with the expansion of the spectrum of action of the individual molecules and the effective prevention of resistance in EOS [62,63]. These studies give promising results for the empirical treatment of EOS in LMICs settings and seem to be suitable for further assessment in clinical trials [62,63].

Overall, the available data highlight that AMR surveillance specific to each geographical region, a significant global commitment to accessible and effective antimicrobials for high-risk newborns and antibiotic stewardship programs for neonatal sepsis are essential [52].

## 3. Late-Onset Sepsis

LOS is mainly due to the horizontal transmission of microorganisms acquired from the environment after delivery (nosocomial or community acquired infection). The etiological agent varies due to the environmental conditions of the hospital, the sanitation of medical personnel, the prevention strategies and geographical area; in addition, the types of pathogen causing LOS in neonates differ between LMICs compared to those in high-income countries [64,65,66]. The most common causative pathogens of LOS in developed settings are Gram-positive bacteria, especially CoNS, which is the main nosocomial agent of LOS, followed by *S. aureus, Enterococcus* species and GBS [64,65,66,67,68,69,70]. Gram-negative bacteria (i.e., *E. coli*, *Klebsiella pneumoniae*, *Pseudomonas aeruginosa*, *Enterobacter* and *Serratia marcenses*) are responsible for approximately a quarter of LOS cases and usually occur more frequently in neonates who underwent central venous access, mechanical ventilation, parenteral nutrition and hospitalization [71,72]. The rate of fungal LOS is more prevalent in premature infants and those who recently received antibiotics, and the most common fungi is *Candida albicans* [71,72]. Viruses are the least-frequent agents attributed to LOS, with herpes simplex viruses being the most frequent [69,70]. While in high-income countries, the most common causes of LOS are Gram-positive bacteria, the evidence from LMICs suggests that LOS is predominantly caused by Gram-negative organisms, of whom the most representative belong to the Enterobacteriaceae group (*Klebsiella pneumoniae*, *E. coli* and *Enterobacter* species), followed by *Pseudomonas aeruginosa* [10,11,12,73,74,75,76,77,78].

The clinical presentation of LOS is typically non-specific, and laboratory investigations lack a negative predictive value to confidently exclude the presence of infection [64,65]. For these reasons, the treatment of LOS can be divided into antimicrobial therapy for suspected (empirical) or known (definitive) pathogens. Consequently, as for EOS cases, most infants who start empirical antimicrobial therapy do not have an underlying infection. Empirical treatment involves the administration of broad-spectrum antibiotics, with the goal of covering the most likely causative pathogens before the definitive culture results are available [64,65,66]. If a culture is positive, pathogen-targeted therapy should be initiated based on the sensitivities [64,65,66].

Consideration of the current epidemiology of LOS, exposures (community or hospitalized status at the onset of LOS), local bacterial prevalence and AMR patterns is crucial to select the most effective and proper antimicrobial combinations for empirical treatment [64,65,66,67,68,69,70]. The duration, dosage and time interval for medications vary depending on the GA, weight, microbe identified, site of infection and the possibility of the antibiotic to penetrate to the site of infection (in case of central nervous system involvement, osteomyelitis or endocarditis) [73]. In general, antibiotics should be discontinued in the absence of the signs and symptoms of infection and when the blood culture is negative.

The common first antibiotic combination used for empiric Gram-positive coverage, the main cause of LOS, is a glycopeptide antibiotic, often Vancomycin, plus an aminoglycoside (e.g., Gentamycin or Amikacin) or an antibiotic with optimal penetration of the cerebrospinal fluid if meningitis is suspected (e.g., Cefotaxime) [32,65,66,68,74]. However, due to increased Vancomycin resistance, narrow empirical first-line therapy with a β-lactam antibiotic (most commonly Ampicillin, Flucloxacillin, Nafcillin or Oxacillin), combined with an aminoglycoside could be initiated in infants who are non-colonized with methicillin-resistant *Staphylococcus* aureus (MRSA) to offer anti-staphylococcal coverage and reduce Vancomycin use in neonatal intensive care units [66,67,75,76,77,78,79,80,81]. In high-income countries, most identified pathogens are susceptible to the empirical antibiotic regimens of β-lactam antibiotic and aminoglycoside, while in LMICs, most of the pathogens isolated from LOS may not be covered by these empirical antibiotics due to the dissemination of resistant bacterial strains, including extended-spectrum beta-lactamase- producing bacteria (ESBL) and MRSA [78,80]. Empirical treatment with Piperacillin–Tazobactam [12,73,75,77,78] and Ampicillin–Sulbactam [66,77], sometimes in combination with or as an alternative to aminoglycoside, is being used increasingly among neonates with LOS in NICUs to cover Gram-positive and Gram-negative beta-lactamase-producing bacteria. Piperacillin–Tazobactam combined with Gentamicin or Meropenem is also effective against *Pseudomonas aeruginosa* [65,82].

An aminoglycoside-based regimen is preferred over Cephalosporin given the reduced risk of resistance, but in the context of the strong clinical suspicion of severe sepsis or Gram-negative meningitis, a third- or fourth-generation Cephalosporin, often Cefotaxime, can be added to the empiric regimen [67,78]. This addition optimizes the therapy against penicillin-resistant Gram-negative organisms and offers enhanced central nervous system penetration. However, the routine empiric use of Cephalosporins is not recommended because of an increased risk for opportunistic *Candida* infection and the high potential for AMR, especially with *Enterobacter* and *Klebsiella* spp.

Infections due to ESBL and AmpC chromosomal beta-lactamase-producing Gram-negative bacteria, such as *Klebsiella* spp. and *E. coli*, require treatment with carbapenems (e.g., Meropenem or Imipenem) due to the high degree of resistance to the commonly used antibiotics, such as Ampicillin, Piperacillin as well as third-generation Cephalosporin [10,69,78,82]. The advantage of Meropenem is its wider antibacterial coverage (i.e., bactericidal activity against *E. coli*, *Klebsiella*, *Enterobacter*, *P. aeruginosa* and against the pathogens responsible for bacterial meningitis) and the possibility of using monotherapy instead of a combination of drugs, but there is critical concern about the choice of carbapenem-resistant Gram-negative organisms (CROs) [69,73,82,83,84].

Treatment with Colistin is available in LMICs for use in case of infections caused by carbapenemase-producing bacteria, but there are only a few studies describing Colistin use in neonates and infants, so it remains the last choice for MDR Gram-negative bacteria after the failure of carbapenems [66,77,78,85]. Furthermore, there are already reports of Colistin-resistant Enterobacteriaceae in neonates [74,86].

The development and diffusion of MDR microorganisms is an important issue in modern neonatology and has been the cause of the decrease in effectiveness of first-line empirical treatments in LOS, supporting the usage of broad-spectrum agents as third- and fourth-generation Cephalosporin, carbapenem, Piperacillin/Tazobactam, Vancomycin and Linezolid (especially in settings where there is a high prevalence of AMR, such as China, India, Pakistan, South Africa and Mexico) [10,11,12,73,74,75]. The use of broad-spectrum antibiotics fosters the spread of MDR pathogens, which then escalates antimicrobial therapy, creating a vicious circle that must be broken by antibiotic policy implementation.

## 4. Conclusions

Considering the impact on morbidity and mortality that EOS and LOS entail, it is essential to start an effective and prompt treatment as soon as possible. Although there are new perspectives on adjuvant therapies, antibiotics are still the most effective tool. The most common antibiotics and combinations are reported in Table 2.

However, as discussed above, the emerging studies reveal important problems, especially in LMICs regarding AMR to first-line antibiotics. The use of targeted antibiotics is peremptory as soon as the pathogen in the culture is detected. Furthermore, the unbridled, preventative use of antibiotics in NICUs is favoring a further significant increase in AMR, as well as brief and long-term health problems for the treated newborns. Based on these results, it should certainly be noted that early therapy is essential, but it should be better assessed whether, when and how to treat neonates with antibiotics, even those at higher risk.

Translational research on endothelial function at the early stages of life, the interactions between the pathogens and their compounds and immune cells, and the effect of endothelial damage on neonatal sepsis may define innovative approaches to endothelium-targeted therapies that may significantly improve the outcomes [87]. Alternative prevention strategies must certainly be implemented, starting with maternal immunization, which has already been shown to protect the newborns from severe infections and currently represents the best defense option against various pathogens [88,89,90,91,92,93,94].

AMR represents a global problem not only in terms of health, but also in terms of health care costs [26]. Considering that we are certainly in the worrying era defined as the “post-antibiotic era”, it is still essential and urgent to define novel strategies for the development of antibacterial compounds with new targets or mechanisms of action. A future strategy could also be to perform well-designed studies to develop innovative algorithms for improving the etiological diagnosis of infections, allowing for more personalized use of the antibiotics to treat EOS and LOS.

## Figures and Tables

**Table 1:** Risk factors and clinical findings to identify newborns who require antibiotic treatment.

Red Flags		Other Indicators (Non-Red Flags)	
Risk Factors	Clinical Findings	Risk Factors	Clinical Findings
Suspected or confirmed infection in previous baby, in case of multiple pregnancy	Apnea Seizures Cardiopulmonary resuscitation Mechanical ventilation Signs of shock	GBS infection in previous baby Maternal GBS colonization, bacteriuria or infection in the current pregnancy Preterm birth following spontaneous labor pPROM > 18 h PROM > 24 h before the onset of labor Intrapartum fever (>38 °C), with suspected or confirmed bacterial infection Clinical diagnosis of chorioamnionitis	Abnormal behavior Abnormal tone Feeding difficulties or feed intolerance Bradycardia or tachycardia Respiratory distress Hypoxia Persistent pulmonary hypertension Jaundice in the first 24 h of birth Signs of encephalopathy Temperature >38 °C, unexplained by environmental factors Excessive bleeding, thrombocytopenia or abnormal coagulation Hypo or hyperglycemia Metabolic acidosis

GBS (Group B *Streptococcus*); pPROM (Preterm Premature Rupture of Membranes).

**Table 2:** Common antibiotics for empirical therapy in suspected neonatal sepsis.

Empirical Antimicrobial Policies	Indication
β-lactam antibiotic + Aminoglycoside (Gentamicin)	Gram-positive and Gram-negative agents; this should be used in neonates non-colonized with MRSA to offer anti-staphylococcal coverage
β-lactam antibiotic + Aminoglycoside (Amikacin)	More resistant Gram-negative and some Gram-positive bacteria (i.e., *Staphylococcus aureus*); this could replace Gentamicin in selected cases (higher-risk preterm neonates or neonates with severe disease)
Glycopeptide + Aminoglycoside	Empiric Gram-positive and Gram-negative coverage; confirmed CoNS and MRSA
Piperacillin + Tazobactam or Ampicillin + Sulbactam	In combination or in alternative to aminoglycoside; Gram-positive and Gram-negative beta-lactamase-producing bacteria
Third- or fourth-generation Cephalosporin	In addition to empiric regimen; for severe penicillin-resistant Gram-negative sepsis or Gram-negative meningitis (no Ceftriaxone)
Carbapenems	ESBL and AmpC chromosomal beta-lactamase-producing Gram-negative; bacterial meningitis
Colistin	CRO

## Data Availability

All the data are included in the manuscript.
